# Supplementary material for: Small-worldness favours network inference in synthetic neural networks
Source: Sci Rep. 2020 Feb 10;10:2296. doi: 10.1038/s41598-020-59198-7 (PMC7010800; doi:10.1038/s41598-020-59198-7)
Supplement: Supplementary file 1 — Supplementary Information. [file 41598_2020_59198_MOESM1_ESM.pdf]

Small-worldness favours network inference in synthetic neural networks

Rodrigo A. García[1,*], **Arturo C. Martí**[1], **Cecilia Cabeza**[1], **and Nicolás Rubido**[1]

[1]Universidad de la República, Instituto de Física de Facultad de Ciencias, Montevideo, 11400, Uruguay.
[*]rgarcia@fisica.edu.uy

ABSTRACT

A main goal in the analysis of a complex system is to infer its underlying network structure from time-series observations of its behaviour. The inference process is often done by using bi-variate similarity measures, such as the cross-correlation (CC) or mutual information (MI), however, the main factors favouring or hindering its success are still puzzling. Here, we use synthetic neuron models in order to reveal the main topological properties that frustrate or facilitate inferring the underlying network from CC measurements. Specifically, we use pulse-coupled Izhikevich neurons connected as in the *Caenorhabditis elegans* neural networks as well as in networks with similar randomness and small-worldness. We analyse the effectiveness and robustness of the inference process under different observations and collective dynamics, contrasting the results obtained from using membrane potentials and inter-spike interval time-series. We find that overall, small-worldness favours network inference and degree heterogeneity hinders it. In particular, success rates in *C. elegans* networks – that combine small-world properties with degree heterogeneity – are closer to success rates in Erdös-Rényi network models rather than those in Watts-Strogatz network models. These results are relevant to understand better the relationship between topological properties and function in different neural networks.

Supplementary Information

Network inference using interspike intervals

The results for the network inference processes shown in Figs. 1 and 2 use the membrane potentials time-series to represent neural behaviour. Alternatively, many studies represent neural behaviour by a reduced time-series, the *inter-spike intervals*[1,2] (ISIs), which are the series of time intervals between consecutive spikes. This is usually carried by arguing that the most relevant information about neural behaviour is coded in this reduced representation of the dynamics. Hence, it is relevant to assess how our results are affected by different representations of neural behaviour. Fig. 5 shows the sensitivity as a function of the coupling strength for Erdös-Rényi, Watts-Strogatz and *C. elegans* networks under the same conditions as Fig. 1, but in this case the cross-correlations are calculated between the ISI series instead of the membrane potentials. Comparing Figs. 5 and 1 we can observe that, in general, the sensitivity is lower when the network inference process is done using the ISIs than when we use the full information from the membrane potentials. Thus, the information reduction implied in the use of the ISIs to represent neural behaviour affects negatively the efficiency of our network inference processes.

Aside the effectiveness of a method to infer the underlying structure of a neural network, we also need to know how robust its results are. In this sense, and in order to gain insight on the differences between the use of ISIs and membrane potentials to perform network inference process, we study how much the inference results change among the different realisations of the topology. Namely, we study the ensemble fluctuations among the results for each network realisation and type of time series used. Firstly, robustness can be assessed by observing the area under the curves in Fig. 1, which is larger for the WS model. Hence, WS networks are better inferred in average for a greater coupling strength range – having similar robustness to the CE network only when $N = 277$. Secondly, robustness can be studied by comparing the standard deviation of the sensitivity, σ_{TPR}, in our $N = 131$ and $N = 277$ ER and WS network ensembles, when we perform inference processes using membrane potentials and ISIs. Fig. 2 shows σ_{TPR} as a function of coupling strengths in the $N = 131$ and $N = 277$ ER and WS network ensembles. We observe that, in all cases, the sensitivity's standard deviation is considerably higher when using membrane potentials than when employing ISIs, for most coupling strengths. Combining these results with those shown in Fig. 1, we conclude that while using the ISIs (instead of the full membrane potentials information) hinders our network inference process, it provides a higher robustness. Hence, there is a trade-off between efficiency and robustness when choosing different neural behaviour representations.

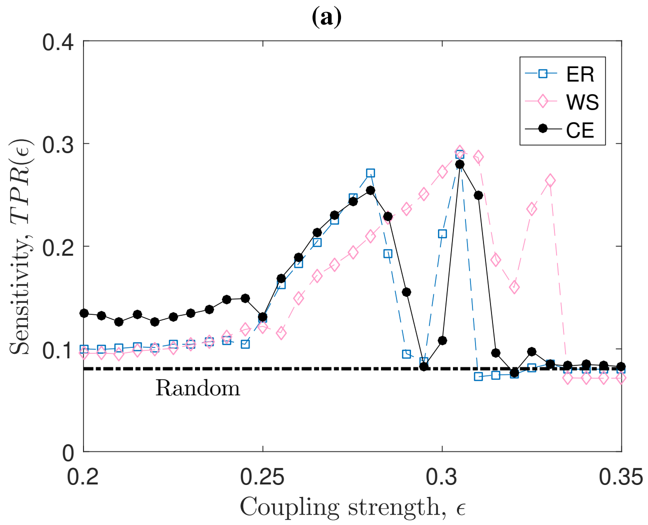

Figure 1. Network inference success rates for different networks, coupling strengths, and sizes, using the interspike intervals time-series. Panel **(a)** [Panel **(b)**] shows the true positive rate, TPR, as a function of the coupling strength, ε, for $N = 131$ [$N = 277$] pulse-coupled Izhikevich maps connected in Erdös-Rényi (ER), Watts-Strogatz (WS), or *C. elegans* (CE) frontal [global] neural networks; with map parameters set such that the isolated dynamics is bursting (see Methods). The TPR values for the ER and WS come from averaging the results over 10 initial conditions and 20 network realisations with similar topological properties to that of the CE (i.e., number of nodes, average degree, and density of connections). For the CE, the results are averaged only on the initial conditions. The TPR in each case is found by the same process as Fig. 1, but using the interspike intervals time-series (considering 2000 spikes) instead of the membrane potentials. The horizontal dashed line in both panels is the random inference TPR, namely, the null hypothesis.

Different time-series length

In all our network inference processes we used a fixed time-series length ($T = 5 \times 10^4$ when using membrane potentials and $T = 2 \times 10^3$ when using interspike intervals). To check the robustness of our results against changes in the length of the time-series used to calculate cross-correlations between pairs of neurons, we calculate the same $TPR(\varepsilon)$ curves shown in Fig. 1, but using different time-series lengths. Fig. 7 shows a comparison between the sensitivity as a function of the coupling strength for Erdös-Rényi networks, using different time-series lengths. We observe a great similarity between all the $TPR(\varepsilon)$ curves, with only quantitative differences when the time-series is too short. We obtain similar results in the Watts-Strogatz and *C. elegans* case. Therefore, we can confirm that our findings are independent of the time-series length considered in our calculations.

ROC Analysis

The analysis of the Receiver Operating Characteristic (ROC) curve is a potent tool for evaluating the efficiency of a classification algorithm[3–5]. It has been used, for example, to assess the efficiency of clinical test for distinguishing between infected and healthy patients[6], or for testing machine-learning-based classification algorithms[7]. In the network inference scene, ROC analysis is often used to test and optimise inference methods[8]. To assess the efficiency of an inference technique, ROC analysis relies on four fundamental quantities: the *Sensitivity*, or *True Positive Rate* (TPR, the fraction of present links that were correctly identified as such), *Specificity*, or *True Negative Rate* (TNR, the fraction of absent links that were correctly identified as such), *False Positive Rate* (FPR, the fraction of absent links that were incorrectly identified as present), and the *False Negative Rate* (FNR, the fraction of existing links that were incorrectly identified as absent).

In the network inference context, these four quantities are naturally related by two constrains: the total number of links ($N[N-1]/2$ in a network with N nodes) and the number M of links conserved by the inference method. It can be readily shown that these constrains relate the four ROC analysis quantities according to Eq. 1.

$$\begin{cases} TPR + FNR = 1 \\ FPR + TNR = 1. \end{cases} \tag{1}$$

Hence, all the information that ROC analysis provides about the inference method is contained in two of the four fundamental

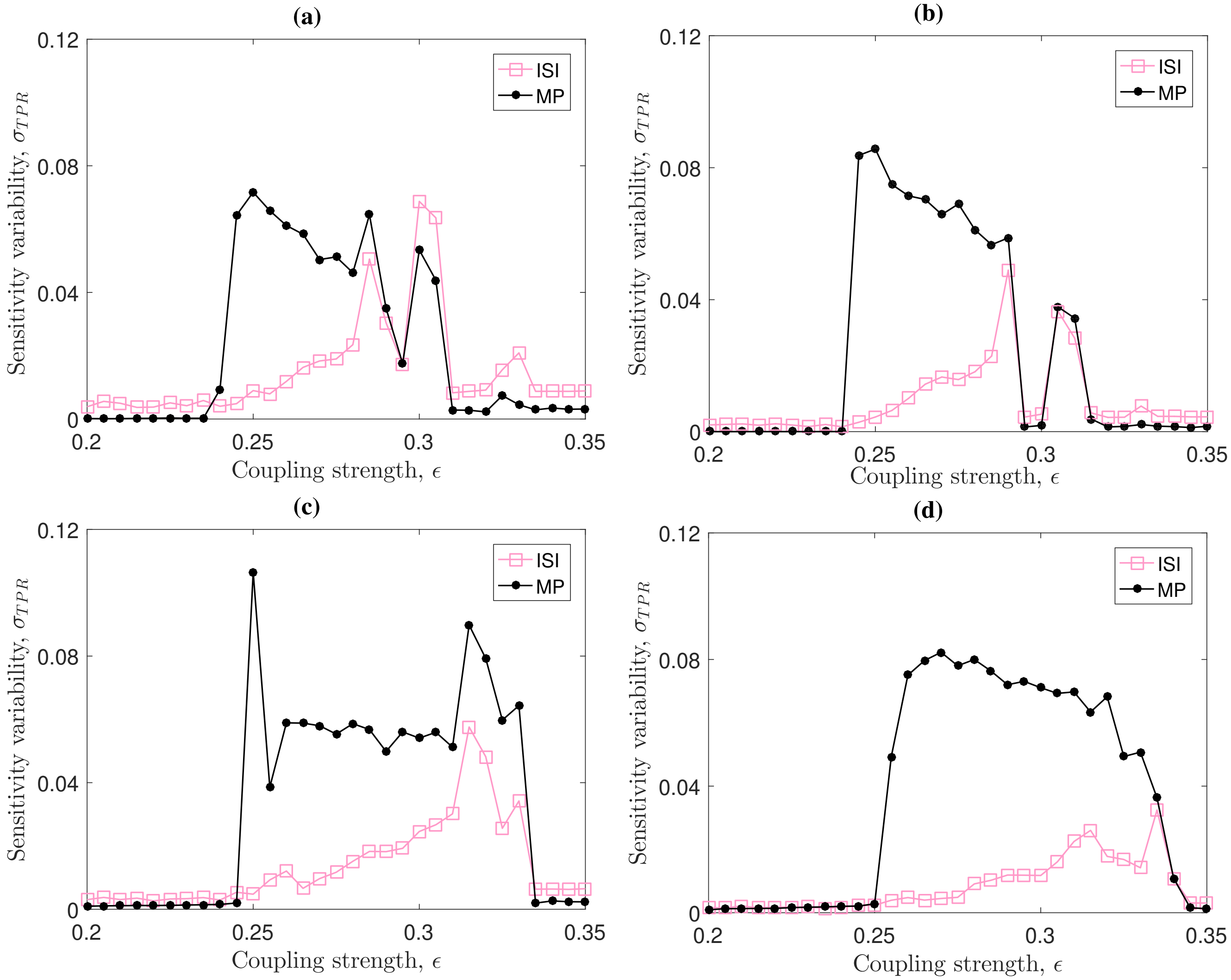

Figure 2. Standard deviation of the sensitivity as a function of the coupling strength in the Erdös-Rényi and Watts-Strogatz network ensembles. In panels **(a)** and **(c)** [**(b)** and **(d)**] we show the standard deviation, σ_{TPR}, as a function of the coupling strength, ε, in the $N = 131$ [$N = 277$] ER and WS network ensembles, respectively. All network ensembles consist of 20 adjacency matrices, and all points result from an average over 10 different initial conditions.

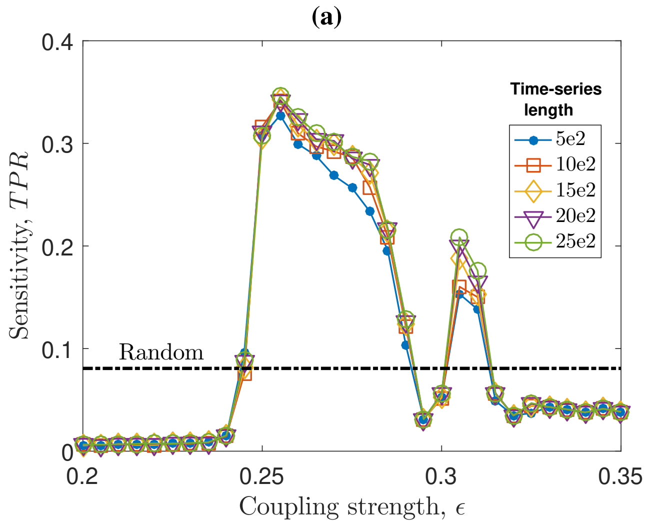

Figure 3. Effect of the time-series length used on the efficiency when inferring ER networks. Panel **(a)** [**(b)**] shows the sensitivity, TPR, as a function of the coupling strength, ε, when inferring $N = 131$ [$N = 277$] ER networks using membrane potentials with different time-series length. All points represent an average among 20 realisations and 10 different initial conditions.

variables. Furthermore, as we have shown in the Results section, if we have an estimate on the link density we can condense all the ROC analysis information in just one variable (e.g. the TPR).

References

1. Sauer, T. Reconstruction of dynamical systems from interspike intervals. *Phys. Rev. Lett.* **72**, 3811 (1994).
2. Reinoso, J. A., Torrent, M. & Masoller, C. Emergence of spike correlations in periodically forced excitable systems. *Phys. Rev. E* **94**, 032218 (2016).
3. Brown, C. D. & Davis, H. T. Receiver operating characteristics curves and related decision measures: A tutorial. *Chemom. Intell. Lab. Syst.* **80**, 24–38 (2006).
4. Fawcett, T. An introduction to roc analysis. *Pattern recognition letters* **27**, 861–874 (2006).
5. Rogers, S. & Girolami, M. A bayesian regression approach to the inference of regulatory networks from gene expression data. *Bioinformatics* **21**, 3131–3137 (2005).
6. Swets, J. & Pickett, R. Evaluation of diagnostic systems: methods from signal detection theory. 1982.
7. Chawla, N. V., Lazarevic, A., Hall, L. O. & Bowyer, K. W. Smoteboost: Improving prediction of the minority class in boosting. In *European conference on principles of data mining and knowledge discovery*, 107–119 (Springer, 2003).
8. Rubido, N. *et al.* Exact detection of direct links in networks of interacting dynamical units. *New J. Phys.* **16**, 093010 (2014).
